# Comparative Genomics of Transcriptional Regulation of Methionine Metabolism in Proteobacteria

**DOI:** 10.1371/journal.pone.0113714

**Published:** 2014-11-20

**Authors:** Semen A. Leyn, Inna A. Suvorova, Tatiana D. Kholina, Sofia S. Sherstneva, Pavel S. Novichkov, Mikhail S. Gelfand, Dmitry A. Rodionov

**Affiliations:** 1 A.A. Kharkevich Institute for Information Transmission Problems, Russian Academy of Sciences, Moscow, Russia; 2 Faculty of Bioengineering and Bioinformatics, Moscow State University, Moscow, Russia; 3 Comprehensive School 463, Moscow, Russia; 4 Lawrence Berkeley National Laboratory, Berkeley, California, United States of America; 5 Sanford-Burnham Medical Research Institute, La Jolla, California, United States of America; University of Groningen, Netherlands

## Abstract

Methionine metabolism and uptake genes in Proteobacteria are controlled by a variety of RNA and DNA regulatory systems. We have applied comparative genomics to reconstruct regulons for three known transcription factors, MetJ, MetR, and SahR, and three known riboswitch motifs, SAH, SAM-SAH, and SAM_alpha, in ∼200 genomes from 22 taxonomic groups of Proteobacteria. We also identified two novel regulons: a SahR-like transcription factor SamR controlling various methionine biosynthesis genes in the Xanthomonadales group, and a potential RNA regulatory element with terminator-antiterminator mechanism controlling the *metX* or *metZ* genes in beta-proteobacteria. For each analyzed regulator we identified the core, taxon-specific and genome-specific regulon members. By analyzing the distribution of these regulators in bacterial genomes and by comparing their regulon contents we elucidated possible evolutionary scenarios for the regulation of the methionine metabolism genes in Proteobacteria.

## Introduction

**Figure 1:**
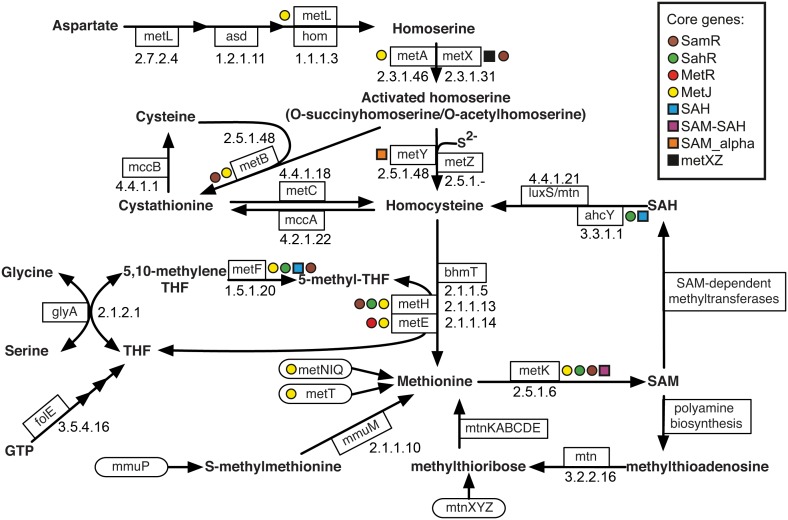
Methionine metabolism and its conserved regulation in Proteobacteria. Genes encoding enzymes for respective reactions are shown in boxes. The Enzyme Commission numbers (EC numbers) of these reactions are shown. Genes encoding transporters of methionine, methylmethionine, and methylthioribose are shown in rounded boxes. Alternative isoenzymes or transporters are shown in different boxes attached to the same arrow. Core regulon members for TFs and riboswitches are shown by colored circles and squares, respectively. The taxonomic distributions of these regulatory interactions are shown in Figure 7.

Methionine biosynthesis in Bacteria involves multiple non-orthologous isofunctional enzymes and biochemical pathway variants (Figure 1) [1]–[3]. It starts from aspartate, which is converted to homoserine by aspartate kinase/homoserine dehydrogenase MetL in *Escherichia coli*. Further activation of homoserine is catalyzed by either homoserine O-succinyltransferase MetA found in *E. coli* [4] and *Pseudomonas aeruginosa* [5], or homoserine O-acetyltransferase MetX, as observed in *Corynebacterium glutamicum* [6] and *Leptospira meyeri* [7]. Interestingly, isofunctional MetA proteins from *E. coli* and *P. aeruginosa* are not homologous [8]. The next pathway step is sulfur incorporation, which can be implemented by either transsulfuration or sulfhydrylation pathways. In the transsulfuration pathway, cysteine plays a role of sulfur donor and homocysteine is formed in two steps by consequent action of cystathionine gamma-synthase MetB and cystathionine beta-lyase MetC [9]. In contrast, the sulfhydrylation pathway uses inorganic sulfide, which is incorporated into homocysteine by O-acetylhomoserine sulfhydrylase MetY [10]. In the final pathway step, homocysteine is methylated to methionine by one of two alternative methionine synthases. The reaction catalyzed by coenzyme B_12_-dependent enzyme MetH is more than 100-fold faster that the reaction catalyzed by B_12_-independent enzyme MetE [11]. For both isozymes, the methyl group is donated by methyl-tetrahydrofolate (methyl-THF), which is formed by methylene-THF reductase MetF. The Gram-positive bacterium *Oceanobacillus iheyensis* has an eukaryotic-type methionine synthase, betaine-homocysteine methyltransferase BhmT [1]. Methionine synthases are generally present both in methionine-synthesizing microorganisms and in methionine auxotrophs, where they are required for the regeneration of S-adenosylmethionine (SAM) [12]. Finally, many microorganisms are capable to directly transport methionine into the cell using specific uptake systems, such as the ATP-dependent ABC-type methionine transporter MetNIQ in *E. coli* [13] and the predicted sodium-dependent methionine permease MetT in *Vibrio* and *Shewanella* spp. [1].

Importance of methionine for the living organisms is not limited to protein biosynthesis, as methionine is a substrate for SAM synthetase MetK. SAM is an essential cofactor in a variety of methylation reactions involved in DNA and RNA metabolism, protein post-transcriptional modifications and other metabolic processes [14]. S-adenosylhomocysteine (SAH) is a product of SAM-dependent methyltransferase reactions and serves as a strong inhibitor of the SAM-dependent enzymes [12], [15]. SAH is converted into homocysteine by one of two recycling pathways. Firstly, SAH can be directly split to adenosine and homocysteine by SAH hydrolase AhcY. Alternatively, SAH is first converted into S-ribosylhomocysteine by SAH nucleosidase Mtn and then utilized to homocysteine and 4,5-dihydroxypentan-2,3-dione by S-ribosylhomocysteine lyase LuxS. SAM is also consumed for the polyamine biosynthesis with formation of methylthioadenosine by SAM decarboxylase. Regeneration of methionine via the 5′-methylthioadenosine salvage pathway was observed in *E. coli* [16] and *B. subtilis* [17].

Genes encoding enzymes and transporters from the methionine pathway are regulated by a variety of RNA and DNA regulatory systems in bacteria. In *E. coli*, methionine metabolism genes are controlled by two specific transcription factors (TFs). SAM-responsive repressor MetJ binds to its operator sites that have a common structure of tandem repeats of two or more MET-boxes with the consensus sequence 5’-AGACGTCT-3’. Comparative genomics analysis of MetJ-binding sites allowed to determine the composition of the MetJ regulons in five taxonomic groups of γ-proteobacteria, *Enterobacteriales, Pasteurellales, Vibrionales Aeromonadales* and *Alteromonadales* [18]. In addition to the methionine biosynthesis and transport gene, the predicted MetJ regulons include cobalamin transporter *btuB* and methionine salvage genes *mtn*. LysR-type transcriptional activator MetR senses homocysteine, a pathway intermediate, and regulates various methionine biosynthetic genes in *E. coli*, *Salmonella* and *Vibrio* spp. [19]–[23]. Recently, a novel SAH-responsive regulator from the ArsR family, named SahR, was shown to control SAM-cycle genes in *Desulfovibrio alaskensis* and other δ-proteobacteria [2]. Gram-positive bacteria from the *Streptococcaceae* family have two LysR-type regulators, methionine-responsive MtaR and *O*-acetylserine-sensing CmbR, that control the methionine and cysteine biosynthetic genes, respectively [24]–[26]. In contrast, TetR-type SAH-responsive repressor McbR regulates both methionine and cysteine biosynthesis genes in actinobacteria from the *Corynebacterium* genus [27], [28].

Various types of *cis*-acting RNA regulatory elements that directly bind SAM or SAH effectors and control gene expression, named riboswitches, were described in many bacterial taxa [29]–[31]. Transcriptional riboswitches commonly have a rho-independent terminator, which is formed when the effector is bound to the riboswitch, leading to termination of transcriptional of downstream genes. Translational riboswitches prevent initiation of translation by sequestering the Shine-Dalgarno sequence via formation of a strong secondary structure, with subsequent inhibition of ribosome binding. The SAM-I (or S-box) riboswitch is widely distributed in Firmicutes, where it controls most of the methionine biosynthesis and transporter genes, as well as SAM synthetase gene *metK* [1]. SAM-II (or SAM_alpha) controls methionine biosynthesis genes *metX*, *metY* and *metA* in α-proteobacteria [32], [33]. The SAH-responsive riboswitch is another widespread type, controlling SAM-cycle genes in several taxonomic groups of proteobacteria [32], [34]. Less abundant riboswitches responding to SAM and/or SAH molecules include SAM-III (or S_MK_-box) that controls *metK* in *Lactobacillus* spp. [32], [35], SAM-IV regulating *metXY* in *Actinomycetales* [32], [36], SAM-V controlling various methionine genes in marine bacterium Candidatus *Pelagibacter ubique* [37], SAM-Chlorobi regulating *metK-achY* genes in *Chlorobiales* [32], [38], and SAM-SAH found upstream of *metK* genes in *Rhodobacterales* [38]. Finally, methionine-specific T-box RNA elements control most methionine transport and biosynthesis genes in the *Lactobacillaceae* family, where SAM riboswitches are much less abundant in comparison to other Firmicutes [32], [39].

In this study, we carried out a comprehensive computational analysis of DNA- and RNA-acting regulatory systems for methionine metabolism in complete genomes of bacteria from 22 taxonomic groups from the phylum Proteobacteria. We report comparative genomics reconstruction of regulons controlled by know methionine-related TFs (MetJ, MetR and SahR) and riboswitches (SAM_alpha, SAH and SAM-SAH). Additionally, we predicted a novel regulator of the methionine and SAM biosynthesis genes in the *Xanthomonadales*, named SamR, and a new RNA regulatory element upstream of the *metXZ* genes in β-proteobacteria. By comparing the metabolic context of the reconstructed methionine regulons, we determined the core, conserved, taxonomy-specific and genome-specific regulon members, and proposed possible evolutionary scenarios for regulation of methionine metabolic genes in Proteobacteria.

## Materials and Methods

A set of 174 complete and 26 draft genomes of Proteobacteria was collected and downloaded from the MicrobesOnline database [40] (Table S1). Closely related strains and species were excluded from the analysis. The obtained 199 representative genomes were classified into 22 taxonomic groups using the phylogenetic species tree in MicrobesOnline [40]. Each of these groups contains 3 to 15 genomes of bacteria from the same taxonomic family or order. Orthologs of the MetJ, MetR and SahR regulators in the representative genomes were identified as bidirectional best hits using blastp (Table S1), and were confirmed by construction of phylogenetic trees using the PhyML software [41]. Paralogs of the MetR and SahR regulators were identified by bidirectional genome-wide similarity searches with 30% of identity threshold using the Smith-Waterman algorithm implemented in the Genome Explorer program [42].

For regulon reconstruction we used the established comparative genomics approach based on identification of candidate TF-binding sites (TFBSs) in closely related bacterial genomes, as previously described [43]–[45]. Briefly, to find conserved TFBS motifs for the MetJ, MetR and SahR regulators in each taxonomic group of Proteobacteria, we used initial training sets of genes that are orthologous to previously established regulon members in model species [2], [18]–[20], [22], [23], [46], and then updated each set by the most likely TF-regulated genes confirmed by the comparative genomics tests as well as the functional considerations (i.e., involvement of candidate target genes in the methionine and SAM metabolism).

The RegPredict Web server (http://regpredict.lbl.gov) [47] allows simultaneous analysis of multiple microbial genomes and integrates information on location of regulatory sites, gene orthologs, operon predictions, and functional gene annotations. Using the Discover Profile tool in RegPredict, we identified common palindromic (for MetR and SahR) or tandem repeat (for MetJ) DNA motifs and constructed respective position weight matrices (PWMs). The initial PWMs were used to scan the studied genomes and identify additional TF-regulated genes and operons that share similar binding sites in their upstream regions. The genome scan parameters were set up to reduce the chance of nonfunctioning sequences from being detected. Specifically, positions of candidate regulatory sites were set between 350 nucleotides upstream and 100 nucleotides downstream of a gene start codon. The maximum intergenic distance for an operon boundary was set to 200 nucleotides under ‘Operon definition’. The conserved regulatory interactions were included in the reconstructed regulogs that are sets of co-regulated genes/operons for which TFBSs are conserved across multiple genomes. False positive TFBSs and genes were eliminated by the consistency check approach [48]. Candidate sites associated with new regulog members were added to the training set, and the respective lineage-specific PWM was rebuilt to improve the search accuracy. Regulatory interactions conserved in less than three genomes per taxonomic group were retained in the reconstructed regulogs if the predicted targets were involved in the methionine metabolism and the associated TFBSs had high scores.

For identification of a TFBS motif for a novel SamR regulator we used the phylogenetic footprinting technique [48] applied to upstream regions of potential target genes/operons involved in the methionine metabolism. Multiple sequence alignments of DNA upstream regions were constructed using the MUSCLE software [49]. To assign the identified TFBS motif to SamR we used the assumption that a local TF has a tendency cluster on the chromosome with regulated genes.

Riboswitches were identified using the covariance models downloaded from the Rfam database [50]. Genomes were scanned using the Infernal program [51]. The identified candidate RNA regulatory sites for each riboswitch family were uploaded into the RegPredict Web server [47] and the respective RNA regulogs were reconstructed using the same approach as for TF regulogs. New regulatory RNAs (potential riboswitches) were found by the phylogenetic footprinting analysis of upstream regions of the methionine metabolism genes in target taxonomic groups of genomes. Secondary structures of two alternative RNA conformations were predicted using Zuker’s algorithm of free energy minimization implemented in the Mfold Web server [52].

To assess conservation of regulatory interactions in the reconstructed orthologous regulogs we calculated the conservation score as the number of gene occurrences in a regulog divided by the number of regulons in a regulog. The average of these taxonomy-specific conservation scores was calculated for all taxonomic groups where the regulated gene was observed (Table S2). For each TF (or riboswitch family), we plotted the average conservation scores for all regulatory targets against the number of taxonomic groups, in which this target was observed as regulated. These plots were used to determine the core, well-conserved, taxon-specific and genome-specific target genes within the analyzed regulons.

The details of all reconstructed TF- and riboswitch-controlled regulogs are deposited in the RegPrecise database (http://regprecise.lbl.gov) [53]. Sequence logos for the derived TFBS motifs were built using the Weblogo 3 package [54]. Biological functions of genes in the reconstructed regulogs were predicted by sequence similarity search against the Swiss-Prot/UniProt database [55], domain architecture analysis in the Pfam database [56], and by using functional gene annotations from the SEED [57] and KEGG [58].

## Results

We selected a set of 199 representative genomes of Proteobacteria and classified them into 22 taxonomic groups by analyzing the phylogenetic species tree (Table 1, Table S1). Using this genome set, we identified orthologs of known TFs involved in the control of methionine biosynthesis, salvage and re-utilization pathways in Proteobacteria (Figure 1), MetJ, MetR and SahR, and reconstructed their regulons using a comparative genomics approach and the RegPredict tool (see Materials and Methods section for details). We further inferred a novel methionine regulator, named SamR, and reconstructed SamR regulons in the *Xanthomonadales* genomes. Then we scanned the genome set using the Rfam profiles for three SAM/SAH-responsive riboswitches and reconstructed their cognate regulons. Finally, we identified and described a novel candidate RNA regulatory element for methionine biosynthesis genes in β-proteobacteria. All reconstructed regulons are described in more detail below.

**Table 1:** Statistics of reconstructed methionine regulons in Proteobacteria.

Taxonomic group^a^	N.G.^b^	MetJ	MetR	SahR/SamR^c^	SAH	SAM_SAH	SAM_alpha	metXZ
γ-proteobacteria								
*Enterobacteriales*	12	12	12					
*Pasteurellales*	9	9	8					
*Vibrionales*	10	10	10					
*Aeromonadales,* and*Psychromonadaceae* 1	6	6	5					
Various *Alteromonadales*	9	9	4					
*Shewanellaceae* 1	16	16	16					
*Oceanospirillales,* and variousγ-proteobacteria	12		12	9				
*Pseudomonadaceae* 2	8		8	8	7			
*Moraxellaceae* 2	4				2			
*Xanthomonadales*	4		3	4^c^	3			
β-proteobacteria								
*Ralstonia* 3	6		6		6			6
*Burkholderia* 3	8		7		8			8
*Comamonadaceae* 3	11		5		11			10
*Alcaligenaceae* 3	3		3					3
Various β-proteobacteria	12		8		11			8
α-proteobacteria								
*Rhizobiales*	15			11			14	
*Rhodobacterales*	15		13	2		13	14	
*Rhodospirillales*	9			7				
*Sphingomonadales*	7			7				
*Caulobacterales*	4			4				
δ-proteobacteria								
*Desulfovibrionales*	10			9				
*Desulfuromonadales*	9			6				
Summary	199	62	120	67^c^	48	13	28	35

The table shows number of genomes containing the methionine regulons per a taxon.

a Genomes are classified into 22 taxonomic groups by analyzing the phylogenetic species tree on the MicrobesOnline [40]. The detailed list of analyzed genomes and taxonomic groups is given in Table S1.

b This column shows the number of analyzed genomes in each taxon.

c This column combines the numbers of SahR and SamR regulons. SamR was identified only in *Xanthomonadales*, whereas SahR regulons are distributed across the remaining nine groups.

1 this family belongs to the *Alteromonadales* order.

2 this family belongs to the *Pseudomonadales* order.

3 this family belongs to the *Burkholderiales* order.

### MetJ regulon

Orthologs of the MetJ repressor were identified in 62 genomes from six taxonomic groups of γ-proteobacteria (Table 1). All analyzed genomes from these taxonomic groups possess a single MetJ ortholog, suggesting MetJ is a universal regulator in these lineages. The MetJ binding motif is well-conserved among all studied taxa (Figure S1). The reconstructed MetJ regulons contain 62 orthologous groups of target genes, of which 44 targets were previously described (Table S2). Among novel predicted targets of MetJ are *btuFCD* (B12 transporter), *thrABC* (threonine biosynthesis) and *serA* (phosphoglycerate dehydrogenase) in *Alteromonadales*; *csd* (cysteine desulfurase) in the *Vibrionales* and *asd* (aspartate semialdehyde dehydrogenase) in *Aeromonadales*.

**Figure 2:**
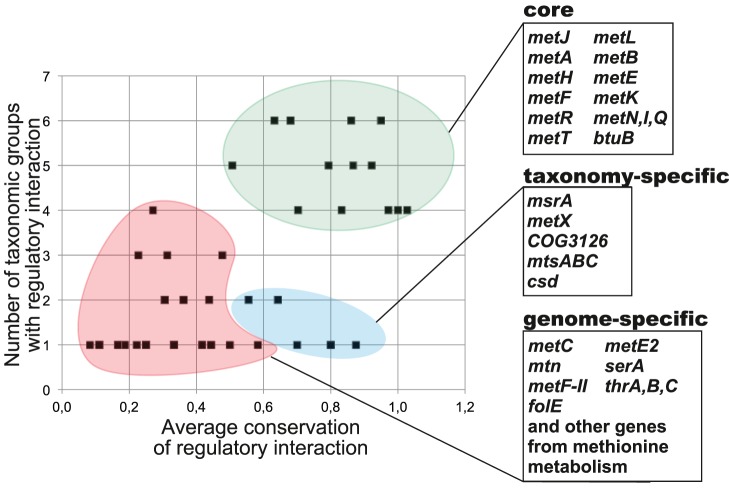
Conservation of regulatory interactions in the reconstructed MetJ regulons. The core regulon, taxon- and genome-specific regulon members are highlighted and listed along with their average conservation scores and functional annotations in Table S2.

By analyzing the average conservation and taxonomic distribution of the predicted MetJ regulatory interactions, we classified the predicted regulon members into three groups: (i) core regulon genes, (ii) taxon-specific genes, and (iii) genome-specific regulated genes (Figure 2). The core regulon includes genes that have MetJ-regulated orthologs in four or more taxa, and that possess high average conservation scores. The core includes the transsulfuration pathway genes (*metL*, *metA*, *metB*, *metH*, *metE*, *metK*, *metF*), two methionine pathway regulators (*metJ, metR)*, two methionine transporters (*metNIQ* and *metT*), and a B12-transporter component (*btuB*). The taxon-specific regulon members are characterized by strong conservation of regulatory interactions restricted to a single taxonomic group. These genes include cysteine desulfurase (*csd*) and a putative ECF-family transporter (*mtsABC*) in *Vibrionales*, homoserine O-acetyltransferase (*metX*) in *Pasteurellales*, and methionine sulfoxide reductase (*msrA*) in *Shewanella* spp. Another conservatively regulated MetJ target is a hypothetical COG3126-family gene, which is co-localized with *metA* in most *Shewanella* spp. The remaining 41 genes were classified as genome-specific members of the MetJ regulon that are characterized by low conservation of regulatory interactions. Most of these genes are involved in the following methionine-related biological processes: methionine biosynthesis (*metY*, *metC*, *metE2*, *metF-II*), uptake and salvage of methylthioribose (*mtnXYZ* and *mtnKABCDE*) and S-methylmethionine (*mmuP* and *mmuM*), the reverse transsulfuration pathway (*mccAB*), and methionine uptake (*metQ2*).

### MetR regulon

**Figure 3:**
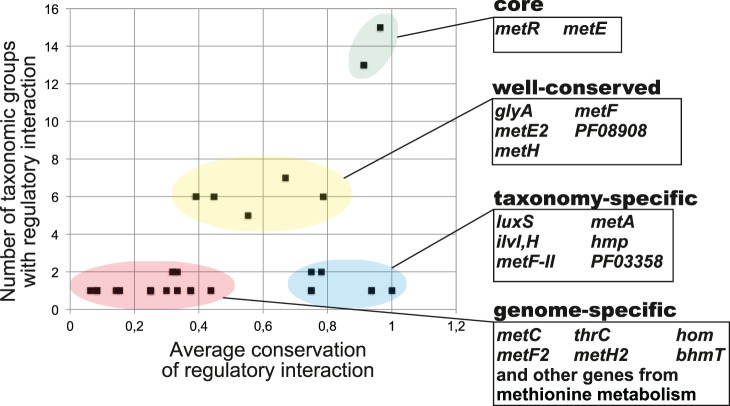
Conservation of regulatory interactions in the reconstructed MetR regulons. The core regulon, taxon- and genome-specific regulon members are highlighted and listed along with their average conservation scores and functional annotations in Table S2.

Orthologs of MetR were found in all studied lineages of γ- and β-proteobacteria except *Moraxellaceae* (Table 1). In addition, MetR orthologs are present in a single lineage of α-proteobacteria, *Rhodobacteriales*. However, despite its widespread taxonomic occurrence, MetR is not ubiquitous within these 15 lineages, being absent in ∼17% of genomes (Figure 1). The MetR-binding motif, a 15-bp palindrome with consensus ATGAA-N_5_-TTCAT, is well-conserved among all studied taxa (Figure S1). The reconstructed MetR regulons contain 31 orthologous groups of target genes, of which only 5 targets were previously described (Table S2). Among novel predicted targets of MetR in *E. coli* and several other Proteobacteria are S-ribosylhomocysteine lyase (*luxS*), which is involved in the SAM cycle, and methionine biosynthesis genes (*metA*, *metF*, *metE2*). Each predicted MetR-regulated gene was assigned to one of the four groups depending on the level of conservation of its regulatory interactions (Figure 3).

The core regulon group includes the *metR* and *metE* genes that are often co-localized and divergently transcribed on the chromosome (thus, they form a divergon). The well-conserved MetR regulon group consists of genes that are regulated in five to seven taxonomic groups (*metH*, *metF*, *glyA*, and *PF08908-metE2*). Therefore, both core and well-conserved MetR regulon groups consist of genes required to support the biochemical conversion of homocysteine to methionine (Figure 1). This observation is in line with the fact that MetR activates genes expression in response to homocysteine [19], [22], [23], thus it can be speculated that the major role of MetR regulons is to eliminate an excess of homocysteine, which is toxic to the cell due to metabolic perturbations of branched-chain amino acid biosynthesis [59].

The taxon-specific regulon group includes genes that are regulated by MetR in one or two lineages and in more than 75% of genomes analyzed within these lineages. Among the taxon-specific regulon members are genes involved in methionine metabolism (*luxS*, *metA*, *metF-II* in *Enterobacteriales* and *Alteromonadales*) and the branched chain amino acid biosynthesis (*ilvIH* in *Shewanella* spp.), as well as a flavohemoprotein (*hmp* in *Enterobacteriales*) and a hypothetical reductase (*PF08908* in *Xanthomonadales*). The *hmp* gene is known to protect cells from nitric oxide, which in turn can affect homocysteine and withdraw it from the methionine biosynthesis [60]. However, it is not clear how cells could benefit from synchronization of the methionine and branched chain amino acid biosynthesis.

The remaining 17 genes in the reconstructed MetR regulons are characterized by low conservation of regulatory interactions. The group of genome-specific MetR regulon members includes various genes involved in the methionine metabolism (*metC* and *mdeA* in *Shewanella*; *bhmT* and *metH2* in *Rhodobacterales*; *hom, thrC* and *metF2* in *Pseudomonadaceae*; *pfl* in *Vibrionales*). Finally, the predicted MetR regulon in β-proteobacteria is much smaller than in γ-proteobacteria and includes only *metE* and *metR* in most genomes.

### SahR and SamR regulons

**Figure 4:**
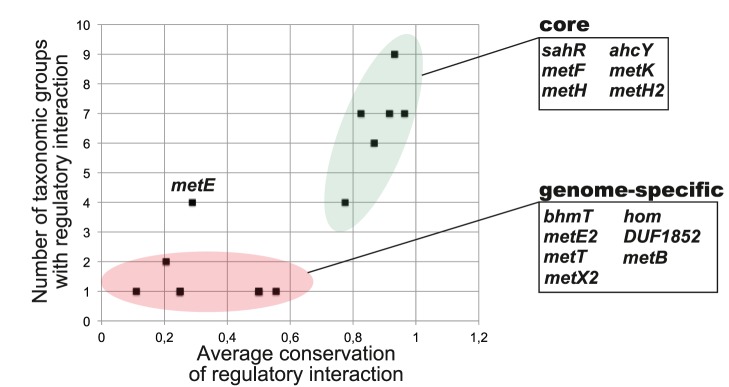
Conservation of regulatory interactions in the reconstructed SahR regulons. The core regulon, taxon- and genome-specific regulon members are highlighted and listed along with their average conservation scores and functional annotations in Table S2.

The SAH-responsive regulators SahR were largely found in α- and δ-subdivisions of Proteobacteria, as well as in two groups of γ-proteobacteria (Table 1) [2]. The SahR-binding motif is a 20-bp palindrome, which is slightly different between α- and δ- proteobacteria, whereas the SahR motifs in γ- and δ-proteobacteria are similar to each other (Figure S1). The reconstructed SahR regulons contain 17 orthologous families of target genes (Table S2) that were classified into two groups based on conservation of their regulatory interactions (Figure 4).

The core regulon consists of genes that were found under SahR regulation in more than 70% genomes that possess SahR and in four and more taxa (Table S2). These genes include the *sahR* regulator itself, as well as the SAM-cycle genes *ahcY*, *metH*, *metH2*, *metF*, and *metK* (Figure 1). The *metE* gene makes an exception as it is distributed in four groups but within these groups it is regulated in 29% of genomes that possess SahR. The genome-specific SahR regulon members include genes that were found in one or two taxonomic groups and are characterized by low conservation of regulatory interactions. Most of genes in this group are involved in the methionine biosynthesis (*bhmT, metE2*, *metX2*, *metB*) and transport (*metT*).

All studied *Xanthomonadales* genomes possess a distant homolog of SahR (∼30% of protein sequence identity), which was named SamR (SAM-dependent Regulator). The *samR* gene in *Xanthomonas* spp. is co-localized with the methionine synthase genes *metH1* and *metH2* and their common upstream regions contain a 24-bp palindromic DNA motif, which partially resembles the SahR-binding motif in γ-proteobacteria (a common consensus ATC-10 nt-GAT) (Figure S1). The reconstructed SamR regulon in *Xanthomonadales* includes other genes involved in the methionine and SAM biosynthesis (*metB*, *metE*, *metF*, *metK, metX2, hom*). The SamR effector remains to be determined. In contrast to the SAM synthetase gene *metK*, other SAM-cycle genes were not found in the SamR regulons, suggesting that SamR responds to SAM rather to SAH.

### Known riboswitch regulons

We used known regulatory RNA motifs from the Rfam database [61] to scan intergenic regions in the analyzed proteobacterial genomes and analyzed the genomic context of candidate regulatory RNAs. Overall, we found 113 riboswitches from three Rfam families (SAH, SAM-alpha, SAM_SAH) that regulate genes from the methionine and SAM metabolism in nine taxonomic groups of Proteobacteria.

**Figure 5:**
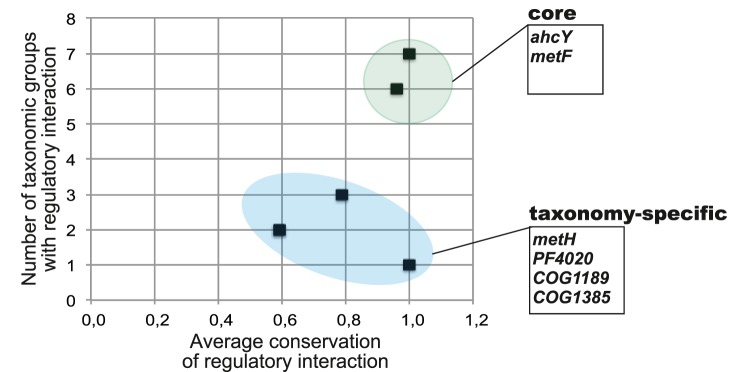
Conservation of regulatory interactions in the reconstructed SAH riboswitch regulons. The core regulon, taxon- and genome-specific regulon members are highlighted and listed along with their average conservation scores and functional annotations in Table S2.

The SAH-responsive riboswitch was found in three lineages of γ-proteobacteria and four groups of β-proteobacteria (Table 1). The content of reconstructed regulons is mostly conserved; individual genomes typically have one or two riboswitch-regulated operons per genome. The core regulon includes the *ahcY* and *metF* genes involved in the SAM cycle (Figure 1). The taxon-specific SAH riboswitch regulon members include a methionine synthase (MetH) and a putative SAM-dependent ribosomal RNA methyltransferase (COG1189) in *Comamonadaceae*, a hypothetical membrane protein (PF04020) in *Ralstonia* spp. and *Burkholderia* spp., and a ribosomal RNA small subunit methyltransferase E (COG1385) in *Moraxellaceae* (Figure 5). In contrast to SahR regulators, SAH riboswitches do not control SAM synthetase *metK*.

**Figure 6:**
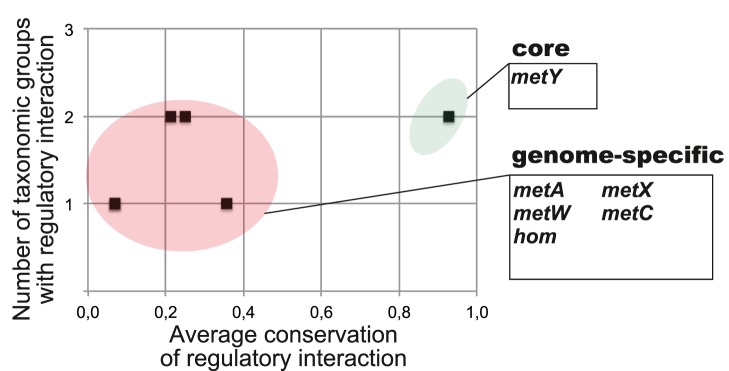
Conservation of regulatory interactions in the reconstructed SAM_alpha riboswitch regulons. The core regulon, taxon- and genome-specific regulon members are highlighted and listed along with their average conservation scores and functional annotations in Table S2.

The SAM_alpha riboswitches were found only in *Rhizobiales* and *Rhodobacterales*. The core regulon includes only one target gene, *metY* (Figure 6). Other target genes were found in less than 40% of regulons in these groups. Among these genome-specific regulon members are genes from the methionine biosynthesis pathway (*hom*, *metA*, *metX*, *metC*), and the *metW* gene that was suggested to facilitate acylation of homoserine [62].

The SAM_SAH riboswitch was found only in *Rhodobacterales* where it controls a single gene, *metK*, in 13 out of 15 studied genomes.

### A novel RNA regulatory element

**Figure 7:**
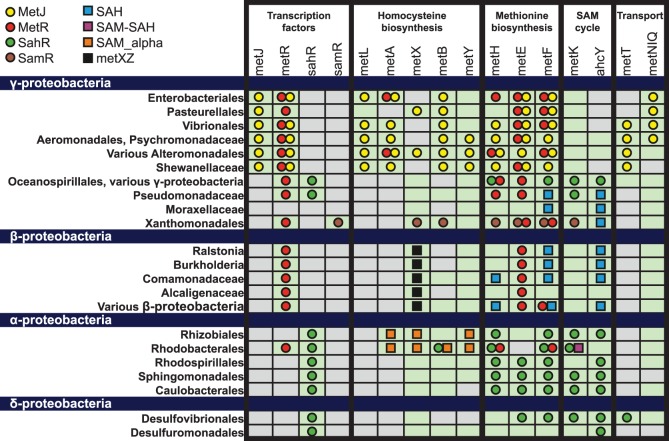
Distribution of regulatory interactions for core members of methionine regulons in Proteobacteria. The presence or absence of gene orthologs in at least one studied genome in a taxonomic group is shown by light green or gray background, respectively. Regulation of at least one gene ortholog within each taxonomic group is shown by colored circles and squares as in Figure 1.

Despite of our knowledge of a variety of methionine regulatory systems, regulation of many methionine biosynthesis and transport genes is still unknown in several taxonomic groups of Proteobacteria (Figure 7). In an attempt to uncover potential transcriptional regulatory mechanisms for these genes, we performed a phylogenetic footprinting analysis of non-coding upstream regions of the selected methionine metabolic genes and found a new putative RNA regulatory element for the *metXW* and *metZ* genes in β-proteobacteria (Figure S2), hence termed metXZ. The *metX* and *metZ* genes constitute the homoserine to homocysteine biosynthesis pathway.

**Figure 8:**
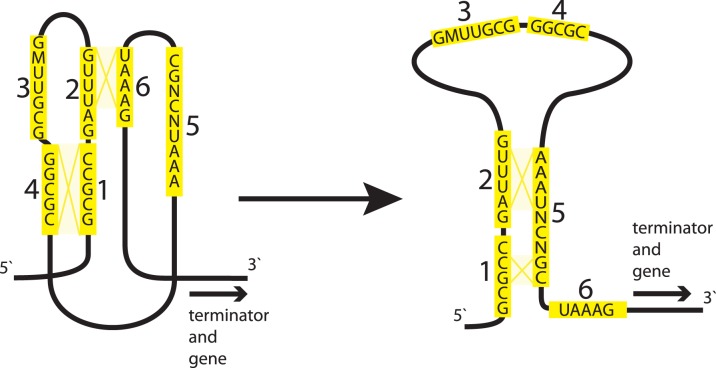
Alternative secondary structures of candidate metXZ RNA element. Regions 1–6 shown in yellow boxes are conserved sequences found in the multiple alignment of leader regions of 34 *metX* and *metZ* genes from β-proteobacteria (Figure S2). In the consensus RNA sequence, N denotes any nucleotide, and M stands for A or C. Possible secondary structures formed by the interaction between the conserved regions are shown by yellow lines.

The metXZ regulatory element contains six highly conserved regions that potentially fold into two alternative RNA conformations (Figure 8). The first conformation could make a pseudoknot structure by interaction of regions 1 and 4, and regions 2 and 6. The second conformation could be a loop with a stem formed by region 5 that interacts with regions 1 and 2. The conserved elements are followed by a potential termination hairpin and a poly-uridine tract. Potential terminators are not conserved on the sequence level but they are consistently present downstream of the metXZ regulatory elements. Interestingly, the highly conserved region 3 does not have any complementary sequence with other regions of the metXZ element. We speculate that the GC-rich regions 3 and 4 could play a role in the regulation of terminator hairpin formation by interaction with one of its stems and formation of an alternative structure of antiterminator.

## Discussion

The results of this study demonstrate considerable variability of the transcriptional regulatory systems for the methionine metabolism genes in Gram-negative bacteria from the phylum Proteobacteria. Three known transcription factors (MetJ, MetR, SahR) and three known riboswitch families (SAH, SAM-SAH, SAM_alpha) appear to control methionine regulons in diverse proteobacterial lineages. We applied comparative genomics approaches for reconstruction of these methionine regulons in ∼200 representative genomes from 22 taxonomic groups of Proteobacteria. We report identification of novel TF and RNA regulons (SamR, metXZ) in several lineages of γ- and β-proteobacteria. Analysis of the distribution of these regulatory systems in bacterial genomes and comparative analysis of reconstructed regulon contents allowed us to elucidate possible evolutionary scenario(s) for the regulation of the methionine pathway.

Among the studied regulons MetJ has the simplest evolutionary history. The distribution of the SAM-responsive TFs in the genomes of γ-proteobacteria suggests that MetJ has possibly arisen in a common ancestor of *Enterobacteriales*, *Pasteurellales*, *Vibrionales, Aeromonadales*, and *Alteromonadales*. The MetJ regulons in all these lineages except *Pasteurellales* possess a highly conserved core including genes involved in the methionine and SAM biosynthesis, methionine transport, and regulation (Figures 1 and 2). Thus, one may speculate that MetJ initially controlled the above core regulon genes, whereas taxon- and genome-specific regulon members were added late in the evolution of MetJ regulons. The *Pasteurellales* group that includes many pathogenic species with reduced genomes differs from other groups by: (i) a unique version of the transsulfuration pathway that utilizes MetX for homoserine activation; (ii) the relatively small size of MetJ regulons with a loss of MetJ binding sites for many core regulon genes, and (iii) appearance of *metX* as taxon-specific regulon member. Overall, in comparison with other methionine regulons, the MetJ regulons contain a relatively large number of target genes (9–33 genes versus 2–12 target genes in the MetR and SahR regulons). The global character of MetJ regulons may potentially explain their strong conservation across γ-proteobacteria, and the absence of potential horizontal transfers of these TFs and their binding sites to other lineages.

Homocysteine-responsive regulators MetR were observed in all studied taxa of β- and γ-proteobacteria except *Moraxellaceae*. However, in some taxa (e.g. *Comamonadaceae* and *Alteromonadales*) MetR-dependent regulation was absent in more than half of genomes (Table 1). The phyletic distribution of MetR orthologs suggests their ancient origin in a common ancestor of β-, γ- and α-proteobacteria. The reconstructed MetR regulons have a highly conserved core containing the *metE* and *metR* genes (Figure 3). Furthermore, the *metF*, *metH*, and *glyA* genes have conserved regulatory interactions in 5–7 taxonomic groups of γ-proteobacteria (Table S2). All these genes are involved in the last step of methionine biosynthesis – conversion of homocysteine to methionine (Figure 1). Interestingly, MetR regulons in *Rhodobacterales* do not contain the core regulon gene *metE,* but instead they have a taxon-specific regulon member *metF*. Thus, the potential evolutionary history of MetR regulons is rather complicated and includes both taxonomy–specific regulon expansions (e.g., acquisition of *hmp*, *luxA* and *metA* in *Enterobacteriales*), contractions (e.g. loss of *metE* in *Alteromonadales*), as well as numerous genome-specific variations in the regulon composition.

SAH-responsive regulator SahR was found in all studied groups of α- and δ-proteobacteria, but in most of these lineages it is not universal (Table 1). In addition, SahR regulators were found in two related lineages of γ-proteobacteria. The reconstructed SahR regulons have a mostly conserved core involved in the methionine synthesis from homocysteine and the SAM cycle (Figure 7). In many proteobacterial genomes SahR regulons are expanded by various genes involved in the methionine biosynthesis (*bhmT*, *hom-metX-metB*), uptake (*metT*) and other metabolic pathways (e.g. *panC* involved in the pantothenate biosynthesis). A distant homolog of SahR identified in *Xanthomonadales*, named SamR, has a larger regulon including the methionine/SAM biosynthesis pathway from homoserine. An interesting, unresolved question is whether SamR retains SAH as an effector molecule (similar to SahR [2]), or it has evolved to respond to SAM.

At least four families of RNA elements regulate the methionine metabolism in Proteobacteria. These include three known families of SAH- and/or SAM-responsive riboswitches and a new conserved RNA element, named metXZ. On average, riboswitches control 1–2 operons per genome and are characterized by mostly conserved regulatory interactions across taxonomic lineages. The observed changes in the riboswitch regulon content were often achieved via likely operon rearrangements. For instance, the SAH riboswitch is located upstream of the *ahcY* genes in all studied genomes from seven lineages, the *metF* genes belong to the *ahcY* operons in all these lineages except *Xanthomonadales*, whereas taxon-specific regulon members PF04020, COG1189, COG1385 are encoded by the same operons only in certain groups of genomes (Table S2). The SAM_alpha riboswitch was found upstream of the *metY* genes in 26 out of 30 genomes from two lineages of α-proteobacteria. Among *Rhodobacterales*, the regulon content was changed in the *Hyphomonadaceae* family to substitute *metY* with either *metA* or *hom-metXC*. In *Rhizobiales*, the SAM_alpha regulon was expanded to include *metA* in six genomes, mostly from the *Rhizobiaceae* family, and *metXW* in five genomes from the *Bradyrhizobiaceae* family. Thus, the evolution of SAM_alpha riboswitches likely involved several independent family-specific duplications. The SAM-SAH riboswitch has the simplest evolutionary history as it has been found only upstream of *metK* genes in *Rhodobacterales*.

The metXZ regulatory element was found in most of the studied genomes of β-proteobacteria in a single copy per genome, suggesting its birth in their common ancestor. In 30 out of 35 genomes, the metXZ element controls the *metXW* genes, however in three *Methylophilales* spp., *Nitrosospira multiformi,* and *Thiobacillus denitrificans* it was found upstream of the *metZ* gene encoding an alternative enzyme from the sulfhydrylation pathway. The comparative analysis of gene neighborhoods of the metXZ element-regulated genes suggests that it was likely re-located from *metX* to *metZ* upstream regions in a common ancestor of these β-proteobacteria.

In addition to numerous taxonomy-related changes in the content of methionine regulons, many genes also change the regulators in small subsets of genomes in the same taxonomic group (Figure 7). The most notable case is the *Rhodobacterales* group that includes the *Hyphomonadaceae* family (two studied genomes), where the SahR regulator substitutes the SAM-SAH and MetR regulators that are conserved in the remaining 13 genomes. In the group of various β-proteobacteria, *metF* is regulated by SAH riboswitch in most of the analyzed genomes, however its regulation has changed to MetR in *Neisseria* spp. In the *Oceanospirillales/Alteromonadales* group, the *metH* gene is regulated by either SahR or MetR. In *Xanthomonadales*, we found two paralogs of *metF* (e.g., XCC0314 and XCC0739 in *Xanthomonas campestris*), that are controlled by MetR and SamR, respectively. In the same group, the *metE* gene is predominantly regulated by MetR, however *Xylella fastidiosa* has lost MetR and acquired SamR-dependent regulation of *metE*.

Both studied SAH-responsive regulons are absent from several lineages of γ-proteobacteria including *Enterobacteriales*, *Pasteurellales*, *Vibrionales*, *Aeromonadales*, and *Alteromonadales* (Figure 7). Two unique features of the methionine metabolism can be attributed to these lineages. First, all these species except two *Idiomarina* spp. lack the SAH hydrolase *ahcY*, but possess a different pathway for SAH recycling that uses the SAH nucleosidase Mtn. Second, the methionine metabolism in these γ-proteobacteria is regulated by the SAM-dependent repressor MetJ. Thus, the presence of either SahR or SAH riboswitch regulon positively correlates with the presence of the SAH hydrolase-dependent pathway of SAH recycling. Indeed, the *ahcY* gene is one of the two most conserved core members of both SahR and SAH riboswitch regulons. Based on the observed genomic context associations we propose that both analyzed SAH-responsive regulons co-evolved with the target *ahcY* genes.

We also observed a number of interconnections between the reconstructed methionine regulons. The most common link between regulons is simultaneous co-regulation of *metE*, *metF*, *metA*, and *metH* by both MetJ and MetR in γ-proteobacteria (Figure 7). Interestingly, these two TFs form numerous feed-forward loops in 43 genomes, when MetJ controls the *metR* gene and both MetR and MetJ co-regulate their targets. Another example of an overlap between the methionine regulons is the *hom-metXC* operon in *Oceanicaulis alexandrii*, which is regulated by a SAM_alpha riboswitch and SahR.

A number of core members of methionine regulons are still not covered by the reconstructed regulon network in several taxa of Proteobacteria (Figure 7). For instance, the *metY*, *metK* and *metNIQ* genes are not regulated by known methionine regulators in β-proteobacteria. We have attempted to identify novel regulatory elements/regulators for these methionine metabolism genes using the comparative genomics approach. However, our attempts were unsuccessful indicating that either regulatory interaction involving these genes are not conserved within their taxonomic groups or these genes are constitutive.

In conclusion, the comparative genomics analysis revealed patterns of genomic distributions and yielded regulon contents for eight methionine-related regulatory systems in diverse lineages of Proteobacteria. Although each particular regulatory interaction predicted here requires experimental verification, the emerging overall picture seems to be consistent and robust.

## Supporting Information

Figure S1 **Predicted DNA-binding motifs of MetJ, MetR, SahR and SamR transcription factors in the analyzed taxonomic groups of Proteobacteria.**
(PDF)

Figure S2 **Predicted RNA regulatory element in upstream regions of the** ***metXW*** **and** ***metZ*** **genes in β-proteobacteria.**
(DOCX)

Table S1 **Distribution of methionine regulators in the studied genomes of Proteobacteria.**
(XLSX)

Table S2 **Taxon-specific and average conservation of regulatory interactions in the reconstructed regulons.**
(XLSX)
