# Supplementary material for: Comparative Genomics of Transcriptional Regulation of Methionine Metabolism in Proteobacteria
Source: PLoS One. 2014 Nov 20;9(11):e113714. doi: 10.1371/journal.pone.0113714 (PMC4239095; doi:10.1371/journal.pone.0113714)
Supplement: Figure S1 — Predicted DNA-binding motifs of MetJ, MetR, SahR and SamR transcription factors in the analyzed taxonomic groups of Proteobacteria. (PDF) [file pone.0113714.s001.pdf]

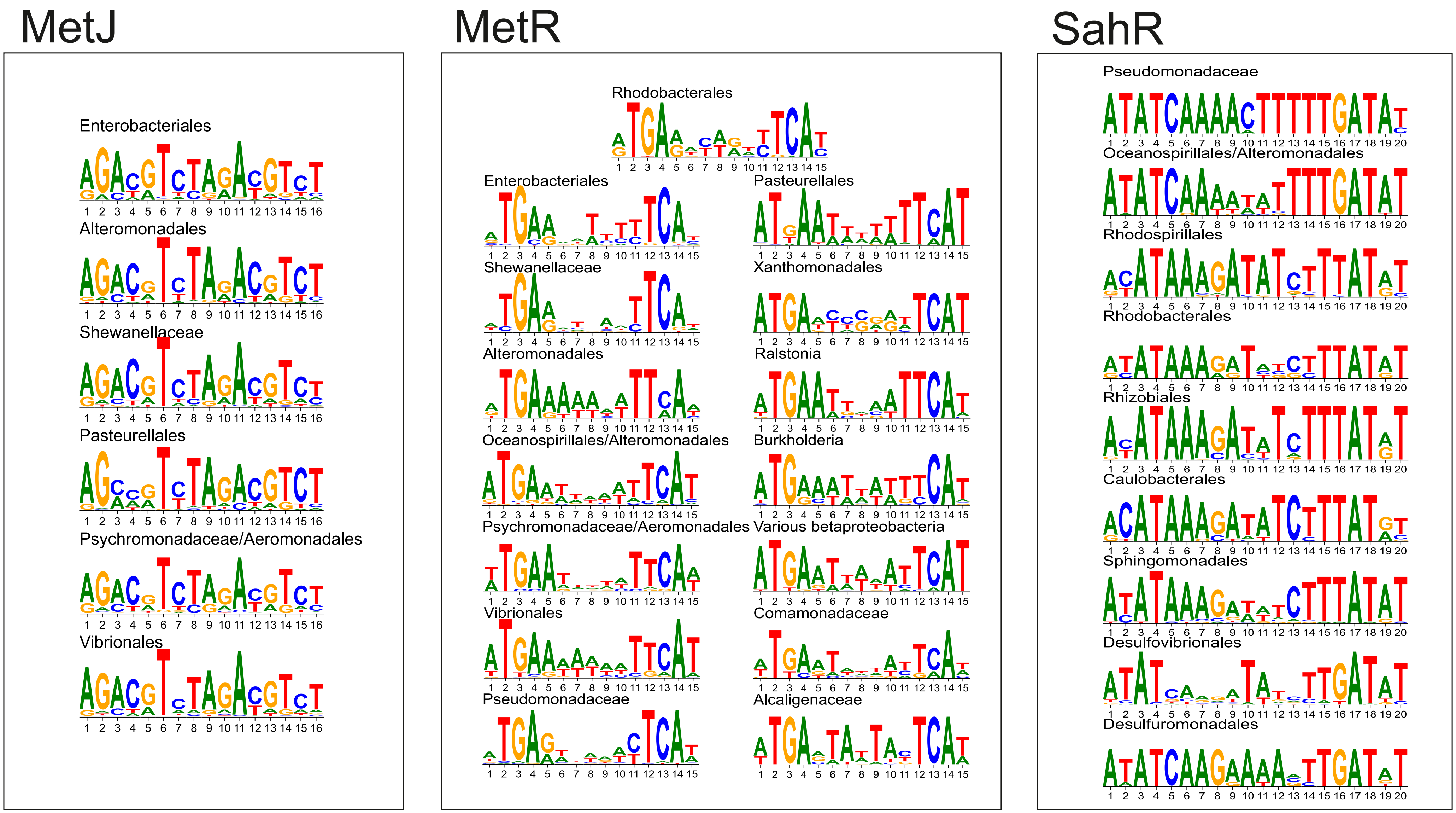

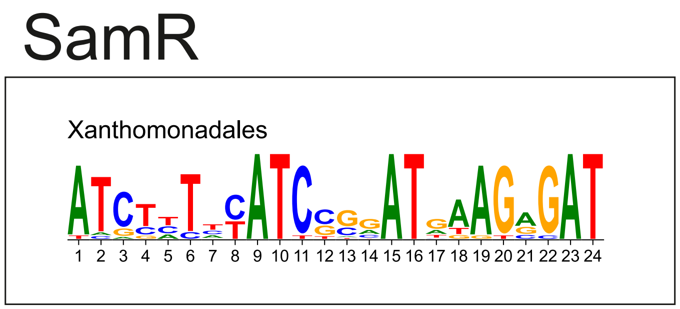

Figure S1. Predicted DNA-binding motifs of MetJ, MetR, SahR and SamR regulators in the analyzed taxonomic groups of Proteobacteria.
