# Supplementary material for: Comparative Genomics of Transcriptional Regulation of Methionine Metabolism in Proteobacteria
Source: PLoS One. 2014 Nov 20;9(11):e113714. doi: 10.1371/journal.pone.0113714 (PMC4239095; doi:10.1371/journal.pone.0113714)
Supplement: Figure S2 — Predicted RNA regulatory element in upstream regions of the metXW and metZ genes in β-proteobacteria. (DOCX) [file pone.0113714.s002.docx]

**Figure S2. Predicted RNA regulatory element found in upstream regions of *metXW* and *metZ* genes in β-proteobacteria.**

The first column and a table below contains genome name, locus_tag, and gene name associated with a RNA element.

Highly conserved regions 1-6 are marked on the top line of the alignment. Complementary parts of regions 1 and 4 and 2 and 6 of the first proposed RNA secondary structure are highlighted in yellow and green, respectively. Complementary parts of regions 1, 3 and 5 of an alternative RNA secondary structure are marked by red font. Grey and dark grey background colors indicate left and right shoulders of the proposed terminator hairpin. The start codon of downstream gene is underlined.

<-1-><--2-> <--3--><-4->

Bxe_A4388 ---------A---UCAAAAACCAUGUAC--GCGCCGAUUUG----------C--------------UGACUCGAUUGCGGGCGCGC--GAACUGCCGGGAAAC----------

Bphy_0061 ---------GCUUGUACAAC----------GCGCCGAUUUG----------C--------------UGACUCGAUUGCGGGCGCGC--GAACCAGCGGAUGAUAUUCAUCCAU

BMA3246 ---------GCUCGGAAAGC----------GCGCCGAUUUG----------C--------------UGACUCGAUUGCGGGCGCGC--GAACCGGCCGGGGCC----------

BPSL0197 ---------GCUCGGAAAGC----------GCGCCGAUUUG----------C--------------UGACUCGAUUGCGGGCGCGC--GAACCGGCCGGGGCC----------

bglu_1g33830 ---------GCCAAGGAAGC----------GCGCCGAUUUG----------C--------------UGACUCGAUUGCGGGCGCGC--GAGCCCUGCCGGACCA---------

Bcep18194_A6450 ---------CAGCCAGAAAC----------GCGCCGAUUUG----------C--------------UGACUCGAUUGCGGGCGCGU--GAACCAGCCGGGGCCUUGAUUCGUC

Bamb_3154 ---------AGGUCGGAAGC----------GCGCCGAUUUG----------C--------------UGACUCGAUUGCGGGCGCGC---GAACCGGCCGGGCCUUGAUGCGUC

Bcep1808_3183 ---------AGGUCGGAAGC----------GCGCCGAUUUG----------C--------------UGACUCGAUUGCGGGCGCGC--GAACCGGCCGGGGCGCGAUGUACGA

Rpic_3723 ---------GCUUCUCUGGC----------GCGCCGAUUUG------------------------ACGACUGGCUUGCGGGCGCGCGGGAGUCCAGGCUGCAC-------GAU

RSc0027 ---------CACUUCCUGAC----------GCGCCGAUUUG------------------------ACGACUGGCUUGCGGGCGCGCGGGAGCCUUGGCCGAUCACCGGUUCGG

Rmet_0143 ---------GCGGGUC--------------GCGCCGAUUUG----------A--------------UGACUGGCUUGCGGGCGCGCGGCGAUGGGAAGGG-------------

Reut_A0186 ---------ACUAUAAUAAGCCGUC-----GCGCCGAUUUG----------A--------------UGACUGGCUUGCGGGCGCGCGGCAGCCGGAUGGUCCCA---------

H16_A0211 ------------------------------GCGCCGAUUUG------------------------AUGACUGGCUUGCGGGCGCGCGGCAGCCAAGGGAC-------------

RALTA_A0154 ------------------------------GCGCCGAUUUG------------------------AUGACUGGCUUGCGGGCGCGCGGCGGCCACGGA------------CCU

MB2181_03730 ---------AUGGAUAAUAUAGAAUGGUUAGCGCCGAUUUG----------------------AAUAAUUCAGCUUGCGGGCGCUC---------------------------

Tbd_1907 ---------UCGGCCGGAC-----------GCGCCGAUUUG----------A--------------AUCCCAGCUUGCGGGCGCUU---------------------------

Bpet0417 ---------AAACUCCUGAACACUUAACCGGCGCCGAUUUG---------CC--------------UGAUCCGCUUGCGGGCGCCU---------------------------

Mmol_0987 GAACUUAGGGCUAACAUGAAAUU-------GCGCUGAUUUGAGUUUGCCAACAUUAAUCAAGGCCACACUCAGCUUGCGGGCGCGU---------------------------

ebA2806 ---------ACGCUAAUUUUUCGCCUCCACGCGCCGAUUUG----------A--------------ACCACAGCUUGCGGGCGCUC---------------------------

Tmz1t_4066 ---------AAGUUCCAGCCACCUA-----GCGCCGAUUUG----------A--------------UCAACAGCUUGCGGGCGCUC---------------------------

Lcho_0321 ------------------------------GCGCCGAUUUG--------------------------AUCCGGCUUGCGGGCGCUC---------------------------

Mfla_1691 ---------AAUAUCAAUCCAU--------GCGCCGAUUUGAGGAGAAGUAG--------------CACUCAGCUUGCGGGCGCAA---------------------------

BAV3187 ---------AGAAUAAGCAUCACAUUGUCGGCGCCGAUUUG---------CC--------------UGAUCCGCUUGCGGGCGCCU---------------------------

Daro_0130 UGUCUGCAGACGCUGAAAGCACUUCAGCG-GCGCCGAUUUG-------------------------AGCUCUGAUUGCGGGCGCUU---------------------------

Mpe_A3584 ------------------------------GCGCCGAUUUG--------------------------AUCCGGAUUGCGGGCGCUC---------------------------

Bpro_4889 ------------------------------GCGCCGAUUUG---------------------------CUCAGCUUGCGGGCGCUU---------------------------

Pnap_4102 ------------------------------GCGCCGAUUUG---------------------------CUCAGCUUGCGGGCGCGU---------------------------

Nmul_A1905 ---------ACCUUCAGGGCAG--------GCGCCGAUUUG----------A--------------UCUUCAGCUUGCGGGCGCAG---------------------------

BB4554 ---------AGAAUUGCGCACAUUGUUCG-GCGCCGAUUUG---------CC--------------UGAUCCGCUUGCGGGCGCCU---------------------------

Aave_4778 ------------------------------GCGCCGAUUUG--------------------------CCACCGCUUGCGGGCGCUC---------------------------

Vapar_5288 ------------------------------GCGCCGAUUUG--------------------------CUUCAGCUUGCGGGCGCUU---------------------------

Ajs_4125 ------------------------------GCGCCGAUUUG---------------------------CACCGCUUGCGGGCGCUU---------------------------

Daci_6044 ------------------------------GCGCCGAUUUG--------------------------CUUCUGCUUGCGGGCGCUU---------------------------

CtesDRAFT_3809 ------------------------------GCGCCGAUUUG--------------------------CCAAAGCUUGCGGGCGCUU---------------------------

**** ****** * **********

<---5---><-6->

Bxe_A4388 -GUUUCCCGC--GGCUCACUAUAAAUGCGGCUAAAGA---GGUCG---------------------------------------------------------------------------

Bphy_0061 CGUCCC------GGUUCACUAUAAAUGCGGCUAAAGA---GGUCG---------------------------------------------------------------------------

BMA3246 -CAUGCCCGGCGGGUUCACUACAAAUACGGCUAAAGA---GGUCG---------------------------------------------------------------------------

BPSL0197 -CAUGCCCGGCGGGUUCACUACAAAUACGGCUAAAGA---GGUCG---------------------------------------------------------------------------

bglu_1g33830 -----CCGGCCGGGUUCACUAUAAAUGCGGCUAAAGA---GGUCG---------------------------------------------------------------------------

Bcep18194_A6450 CUUGUCCCGGC-GGUUCACUACAAAUGCGGCUAAAGA---GGUCG---------------------------------------------------------------------------

Bamb_3154 UUGGUCCCGGCAGGUUCACUACAAAUGCGGCUAAAGA---GGUCG---------------------------------------------------------------------------

Bcep1808_3183 GCGACCCGGC--GGUUCACUACAAAUGCGGCUAAAGA---GGUCG---------------------------------------------------------------------------

Rpic_3723 UGUGGCUCUGGGCUCUCAUUACAAAUGCGGCUAAAGA---GGUUCGGUCAGAGCGGGGGUGGUGAAAGCAAGCGCCCCACGUGCGCCACAUGAGAAC-----------------------

RSc0027 UGUGUGCCCGGGCUCUCAUUACAAAUGCGGCUAAAGA---GGUUCGGUCAGAACGGGGGCAUGAGUCCGCCGUGCGCCACACAGAGC---------------------------------

Rmet_0143 -AAGUCUUCCGGUUGCCAAUAUAAAUGCGGCUAAAGA---GGUUGGGUCGGUGCCUCGCGGAAGAUUCAUGCAUGGCACCGGCACGCGGGACACCGCGAACGC-----------------

Reut_A0186 -CGAUCCUCCGUCUGCGAAUAUAAAUGCGGCUAAAGA---GGUUGGGUCGGCGCCCCCAGGCAGGAUCGGGAUUCACGCCGGCACGCGGAACACCGCGAAUGC-----------------

H16_A0211 -CAAGUUCCGGGUUGCCACUACAAAUGCGGCUAAAGA---GGUUGGGUCGGCGCCCCAUGCCUCCACGGUAGUGGAUGGCGCCGGCACGCGGAAUCCCGCAAGGAAUCCAGUGAAUC---

RALTA_A0154 GGGUCCUGGGGCCUGCCACUAUAAAUGCGGCUAAAGA---GGUUGGGUCGGCGCCCCAUGCCACCACGGCAGUGGAUGGCGCCGGCA-GCGGAAUCCCGCAAGGAAUCCAGCGAAUC---

MB2181_03730 -------------------UAAAAAU-CAGCUAAAGC---GGG-----------------------------------------------------------------------------

Tbd_1907 -------------------GAAAAAUACGGCUAAAGC---GAG-----------------------------------------------------------------------------

Bpet0417 ------------------CUAUAAAUCCAGCUAAAGA---GGUCCGUAUGACCACUCCCGUGCCUGUUCCGCCACAGUCC--------------CCGGGCGUAGAUUCGGCCGGACGCGC

Mmol_0987 -------------------UAUAAACACAGCUAAAGC---GAGUAC--------------------------------------------------------------------------

ebA2806 -------------------AAUAAAUCCGGCUAAAGC---GAGGGCGAC-----------------------------------------------------------------------

Tmz1t_4066 -------------------AACAAAUACGGCUAAAGC---GAGG----------------------------------------------------------------------------

Lcho_0321 -------------------UAGAAAUGCCGCUAAAGA---GGUACAC-------------------------------------------------------------------------

Mfla_1691 -------------------UAUAAAUACUGCUAAAGC---GAGUUGAUA-----------------------------------------------------------------------

BAV3187 -----------------CUUAUAAAUCCAGCUAAAGA---GGUCUGCAAUGACCAGUCCUGCUUUGACCUUGACGCCGGCCCAUGGCGGGACUAAUAUCAAUCCAUGCGCCGAUUUGAGG

Daro_0130 --------------------AUAAAUACAGCUAAAAC---GGGAA---------------------------------------------------------------------------

Mpe_A3584 -------------------AACAAAUGCAGCUAAAGA---GGGGCCC-------------------------------------------------------------------------

Bpro_4889 -------------------AAAAAGUACCGCUAAAGA-----------------------------------------------------------------------------------

Pnap_4102 -------------------AAAAAGUACCGCUAAAGA---CGACUCUGCA----------------------------------------------------------------------

Nmul_A1905 -------------------UAUAAAUUCAGCUAAAGC---GGG-----------------------------------------------------------------------------

BB4554 -----------------CUUAUAAAUCCAGCUAAAGA---GGUCUGAAUGACCAAUCCUGUCCUGAAUUGAAGAUUUGACAAAAGCAGGUACCGAGCGUACCUUCAGGGCAGGCGCCGAU

Aave_4778 -------------------UAUAAAUCCAGCUAAAGACCCGACCGUUGGUGCAGUGCG--------------------------------------------------------------

Vapar_5288 -------------------AAUAAAUUCAGCUAAAGA--CAGUCCAGUUGGCGAACAA--------------------------------------------------------------

Ajs_4125 -------------------UACAAAUCCAGCUAAAGA---CGUGA---------------------------------------------------------------------------

Daci_6044 -------------------UAUAAAUCCUGCUAAAGA---CGACACCAG-----------------------------------------------------------------------

CtesDRAFT_3809 -------------------UAUAAAUCCAGCUAAAGA---CCCGUCCGCGAAGUGACAGUACA---------------------------------------------------------

** * ******

Bxe_A4388 -----------UCAGCCGC---------------------------GCACACA--------------GUCAUUCUCUUCCGUGC-UUCGCCGACG---CCA-------UCUAGCCGCCC-

Bphy_0061 -----------UCAGCCGC---------------------------GCACAU-------------------UGUCCUUCCGCGCAAUCGCCGACACCA----------UUUAGCCGCCC-

BMA3246 -----------UCAGCCUG---------------------------CCCC---------------------CCGCUUUCCGCGC-ACGCCCGACAACCCUG-------UUUAGCCGCCC-

BPSL0197 -----------UCAGCCUG-------------------------------------------------CCCCCGCUUUCCGCGC-ACGCCCGACAACCCUG-------UUUAGCCGCCC-

bglu_1g33830 -----------UCAGCCGC---------------------------GCCAU--------------------CGCUCUUGCGCGC-AUCGCCGACAC---CG-------UCUAGCCGCCC-

Bcep18194_A6450 -----------UCAGCCGC---------------------------GCAUC--------------------UCCGACUCCGCGC-AUCGCCGACACCA----------UUUAGCUGCCC-

Bamb_3154 -----------UCAGCCGC---------------------------GCACC--------------------UUCGCCUCCGCGC-AUCGCCGACA---CCG-------UUUAGCCGCCC-

Bcep1808_3183 -----------UCAGCCGC---------------------------GCAAU--------------------UUCGCCUCCGCGC-AUCGCCGACA---CCG-------UUUAGCCGCCC-

Rpic_3723 -----------CCGAUUGG----------------------------------------------------UUGCGUGAACAGC-GUUGCCGGU-----CGGGUU---UUUUAUUU----

RSc0027 -----------CCGAUUGG----------------------------------------------------UUGCGUUGUCAGC-GUUGCCGGU-----CGGGUU---UUUUCUUU----

Rmet_0143 -----------CCGACUUG---------------------------GCUGCG-------------------AAGCAUCACACGC-ACUGCCAGU-----CGGGUU---UUUUUAUGCCU-

Reut_A0186 -----------CCGACUUG---------------------------GCUGCG-------------------ACGCUUCACACGC-ACUGCCGGU-----CGGGUU---UUUUUAUGCCU-

H16_A0211 -----------CCGACUUG---------------------------GCUGCG-------------------ACGCUUUAUACGCAAAUGCCGGU-----CGGGUU---UUUUUAUGCCC-

RALTA_A0154 -----------CCGACUUG---------------------------GCUGCG-------------------ACGCUUGAUACGCAAAUGCCGGU-----CGGGUU---UUUUUAUGCCC-

MB2181_03730 -----------UAAACCCA---------------------------------------------------------------UCAGCAGCUGUU-----UGGGUU---UUUUUUUGUCU-

Tbd_1907 -----------GCGACGGA----------------------------------------------------AACCCCGCGGCGCGCUUGGCGUU-----CGGGGU---UUUUUCUU----

Bpet0417 CGAUUUGAAUCCCAGCUUGCGGGCGCUUGAAAAAUACGGCUAAAGCGAGGCGA--------------CGGAAACCCCGCGGCGCGCUUGGCGUU-----CGGGGU---UUUUUCUU----

Mmol_0987 -----------ACAAAGAA----------------------------------------------------CCCGUUUUAGCGCAAUGCUUAAG-----CGGGUU---UUUUUAUGGCGC

ebA2806 -----------CCAGUCCG------------------------------------------------------CGUUUCCCAGC------CGGC-----UGGGUU---UUUUGUCUACA-

Tmz1t_4066 -----------GCACCCAG---------------------------UCCGCG------------------------------UUUGCAGCCGGC-----UGGGUU---UUUUGUUUUGC-

Lcho_0321 -----------CCGAUCAA----------------------------------------------------UCCGGUGUCGGCCUCUCGACGGCGCCGCCGGGUU---UUUUGCUUUUC-

Mfla_1691 -----------UUGAUAAA---------------------------GACCCGUU-----------------UUAGCCCAAGCGCUACAG----------CGGGUU---UUUUUAUGCCCG

BAV3187 AGAAGUAGCACUCAGCUUGCGGGCGCAAUAUAAAUACUGCUAAAGCGAGUUGAUAUUGAUAAAGACCCGUUUUAGCCCAAGCGCUACAG----------CGGGUU---UUUUUAUGCCCG

Daro_0130 -----------CCACCCAG--------------------------------------------------------UUCGCGUUUUUCAGCCAAC-----UGGGUU---UUUGUUUUCAG-

Mpe_A3584 -----------UCGACCGU---------------------------UCACCACCGU---------------CACCCGGCCUCGCUAGCG-CAGCGACGCCGGGUUGUCUUUUUCUU----

Bpro_4889 -CGAACAUCCAUGAACCCG---------------------------GACUCG-------------------CCCUUAGCGAUGC---------------CGGGUU---UUCUUUUUGCC-

Pnap_4102 -----------CGAACCCG---------------------------GACUCG-------------------CUUUCAGCGAUGC---------------CGGGUUUUCUUUUUGCUCCAU

Nmul_A1905 -----------AAAACCCG--------------------------------------------------------UUUUUUAGCGAAAGCUGAA-----CGGGUU---UUUUAUUGGGA-

BB4554 UUGAUCU----UCAGCUUGCGGGCGCAGUAUAAAUUCAGCUAAAGCGGGAAAA------------------CCCGUUUUUUAGCGAAAGCUGAA-----CGGGUU---UUUUAUUGGGA-

Aave_4778 -----------ACGACCCG---------------------------GCCUCG-------------------UUUUUAGCGAUGC---------------CGGGUU---UUUUCAU-----

Vapar_5288 -----------GCAACCCG-------------------------------------------------------------GCUC-GUUUUUGGCGAGGCCGGGUU---UUUUC-------

Ajs_4125 -----------CCGAUCGA--------------------------CACGCCU-------------------GCCACCCCAGCACGCACCCGGACCCGGCCUCGUU---GUUUAGCG----

Daci_6044 -----------AGAACCCG---------------------------GCCUCG-------------------UUUUUAGCGAUGC---------------CGGGUU---UUUUCAUGUCCU

CtesDRAFT_3809 -----------CCGACCCG---------------------------GCCUCG-------------------UUUUUAGCGAUGC---------------CGGGUU---UUUUCAU-----

Bxe_A4388 ----------UGUUUUGUUAAAUGGCGGAAUGAAUGGA---------------------AUCGAUCGGUAUCGUC---------------------

Bphy_0061 -------------CAUGUCAGAAGGCGGAAUGAAUGGA---------------------AUCGAUCGGGAUCGUC---------------------

BMA3246 ---------------AGCCAUAAGGCGGAACGAAUGGA---------------------AUCGAUCGGCGUCGUC---------------------

BPSL0197 ---------------AGCCAUAAGGCGGAACGAAUGGA---------------------AUCGAUCGGCGUCGUC---------------------

bglu_1g33830 ---------------AGUCAUGAGGCGCAACGAAUGGA---------------------AUCGAUCGGCAUCGUC---------------------

Bcep18194_A6450 ---------------AGUCAUAAGGCGGAACGAAUGGA---------------------AUCGAUCGGCAUCGUC---------------------

Bamb_3154 ---------------AGUCAUAAGGCGGAACGAAUGGA---------------------AUCGAUCGGCAUCGUC---------------------

Bcep1808_3183 --------------CAGUCAUAAGGCGGAACGAAUGGA---------------------AUCGAUCGGCAUCGUC---------------------

Rpic_3723 ---------------GGUGCC------------AUGACAGAC---------------------AUCCAGGCUGAC---------------------

RSc0027 ---------------GGCGCC------------AUGACAGAAC---------------------UCCAGGUGGAU---------------------

Rmet_0143 ---------------GCCUCACUCGCGGGCCCCGCUCAUG------------------------ACCGAUGUCGCUCCUCCC---------------

Reut_A0186 -----------------GCUUCGCGCGGGCCGAUUCCAUG------------------------ACAGAUGUCGCCUUGCCG---------------

H16_A0211 ------------GUUCGCCACGGGCCGGAUUCCAUGAC---------------------------UGAUGUCGCCCUGCCG---------------

RALTA_A0154 ------------GUUCGCCACGGGCCGGAUUCCAUGAC---------------------------UGAUGUCGCCCUGCCG---------------

MB2181_03730 -----------------AAAUUAGGUAAAGUGAAUAAA--------------------------------UCGUAAUGAAUAAAUUACCAAAUAAA

Tbd_1907 ------------UUGGACUCGAAGAUGAACGACGCAUA------------------------------CGACAUC---------------------

Bpet0417 ------------UUGGACUCGAAGAUGAACGACGCAUA------------------------------CGACAUC---------------------

Mmol_0987 AAAAAAAUGGCGUUGA--CUAAAAGUAGCUGUAAUCAA---------------------GAAUUUAGAGGUAAUGAUGAAUAAUAAUUUACAAUUU

ebA2806 ------------------------GCGAACAAUAUGAU---------------------CCAACCCCAAUCCGUC---------------------

Tmz1t_4066 -------------------AGAUGACGAAAAUGAUCGCACCC------------------------------------------------------

Lcho_0321 --------------GGGAUCCAUGGUGACA------------------------------------------------------------------

Mfla_1691 UCCUUUUUGUCAUUGAGCCCGAGAGCGGGCGGGAAGGU--------------------AAGAUGUAUGAGUCAGCAUGAAUGGCAU----------

BAV3187 UCCUUUUUGUCAUUGAGCCCGAGAGCGGGCGGGAAGGU--------------------AAGAUGUAUGAGUCAGCAUGAAUGGCAU----------

Daro_0130 --------------GAACAGCAUGCCAGGACAAUC---------------------------------CGUCGGC---------------------

Mpe_A3584 --------------GGGUUGAAUAUCGGGAUGGCUUCG----------------------------------------------------------

Bpro_4889 ----------GUUUCACUUUCCGGUCAGUCCGUCUCCAGUCUUGUUCCGAUCACGGGUCUUCUUAUUGUGUCUUCU--------------------

Pnap_4102 CGUUUUCCUAUCGUGGAUCCAUCAACAGUGCCUAUUGC---------------------CCCCUUCCUUGUCGCCUCGCCAGCGUCCAUGCACUUU

Nmul_A1905 ----------AUUUGAGGGAGAUGUCGG---GCAUGUC------------------------------UGACGAU---------------------

BB4554 ----------AUUUGAGGGAGAUGUCGG---GCAUGUC------------------------------UGACGAU---------------------

Aave_4778 -----------------------------------------------------------------CCAUGUCGUUC--------------------

Vapar_5288 ---------------------------------------------------------------AUUGAUGUCGUCA--------------------

Ajs_4125 ---------------------AAGCCGG-------------------------------GUUUUUUCAUGUCGUUC--------------------

Daci_6044 UC----------------------------------------------------------------------------------------------

CtesDRAFT_3809 -------------------------------------------------------------------AUGUCCUUC--------------------

| Organism name | First gene  locus tag | Target gene/operon | Taxonomic Group |
| --- | --- | --- | --- |
| *Bordetella avium 197N* | BAV3187 | *metXW* | Alcaligenaceae |
| *Bordetella bronchiseptica RB50* | BB4554 | *metXW* |  |
| *Bordetella petrii DSM 12804* | Bpet0417 | *metXW* |  |
| *Burkholderia cepacia AMMD* | Bamb_3154 | *metXW* | Burkholderia |
| *Burkholderia glumae BGR1* | bglu_1g33830 | *metXW* |  |
| *Burkholderia mallei ATCC 23344* | BMA3246 | *metXW* |  |
| *Burkholderia phymatum STM815* | Bphy_0061 | *metXW* |  |
| *Burkholderia pseudomallei K96243* | BPSL0197 | *metXW* |  |
| *Burkholderia sp. 383* | Bcep18194_A645 | *metXW* |  |
| *Burkholderia vietnamiensis G4* | Bcep1808_3183 | *metXW* |  |
| *Burkholderia xenovorans LB400* | Bxe_A4388 | *metXW* |  |
| *Acidovorax avenae subsp. citrulli AAC00-1* | Aave_4778 | *metXW* | Comamonadaceae |
| *Acidovorax sp. JS42* | Ajs_4125 | *metXW* |  |
| *Comamonas testosteroni KF-1* | CtesDRAFT_3809 | *metXW* |  |
| *Delftia acidovorans SPH-1* | Daci_6044 | *metXW* |  |
| *Leptothrix cholodnii SP-6* | Lcho_0321 | *metXW* |  |
| *Methylibium petroleiphilum PM1* | Mpe_A3584 | *metXW* |  |
| Polaromonas naphthalenivorans CJ2 | Pnap_4102 | *metXW* |  |
| *Polaromonas sp. JS666* | Bpro_4889 | *metXW* |  |
| *Variovorax paradoxus S110* | Vapar_5288 | *metXW* |  |
| *Cupriavidus taiwanensis* | RALTA_A0154 | *metXW* | Ralstonia |
| *Ralstonia eutropha H16* | H16_A0211 | *metXW* |  |
| *Ralstonia eutropha JMP134* | Reut_A0186 | *metXW* |  |
| *Ralstonia metallidurans CH34* | Rmet_0143 | *metXW* |  |
| *Ralstonia pickettii 12J* | Rpic_3723 | *metXW* |  |
| *Ralstonia solanacearum GMI1000* | RSc0027 | *metXW* |  |
| *Azoarcus sp. EbN1* | ebA2806 | *metXW* | Various  betaproteobacteria |
| *Dechloromonas aromatica RCB* | Daro_0130 | *metXW* |  |
| *Thauera sp. MZ1T* | Tmz1t_4066 | *metXW* |  |
| *Methylobacillus flagellatus KT* | Mfla_1691 | *metZ* |  |
| *Methylophilales bacterium HTCC2181* | MB2181_03730 | *metZ* |  |
| *Methylotenera mobilis JLW* | Mmol_0987 | *metZ* |  |
| *Nitrosospira multiformis ATCC 25196* | Nmul_A1905 | *metZ* |  |
| *Thiobacillus denitrificans* | Tbd_1907 | *metZ* |  |
